# The anuran vocal sac: a tool for multimodal signalling

**DOI:** 10.1016/j.anbehav.2014.07.027

**Published:** 2014-11

**Authors:** Iris Starnberger, Doris Preininger, Walter Hödl

**Affiliations:** a University of Vienna, Department of Integrative Zoology, Wien, Austria; b Vienna Zoo, Wien, Austria

**Keywords:** chemical signal, functional morphology, multimodal signals, visual signal, vocal sac diversity

## Abstract

Although in anurans the predominant mode of intra- and intersexual communication is vocalization, modalities used in addition to or instead of acoustic signals range from seismic and visual to chemical. In some cases, signals of more than one modality are produced through or by the anuran vocal sac. However, its role beyond acoustics has been neglected for some time and nonacoustic cues such as vocal sac movement have traditionally been seen as an epiphenomenon of sound production. The diversity in vocal sac coloration and shape found in different species is striking and recently its visual properties have been given a more important role in signalling. Chemosignals seem to be the dominant communication mode in newts, salamanders and caecilians and certainly play a role in the aquatic life phase of anurans, but airborne chemical signalling has received less attention. There is, however, increasing evidence that at least some terrestrial anuran species integrate acoustic, visual and chemical cues in species recognition and mate choice and a few secondarily mute anuran species seem to fully rely on volatile chemical cues produced in glands on the vocal sac. Within vertebrates, frogs in particular are suitable organisms for investigating multimodal communication by means of experiments, since they are tolerant of disturbance by observers and can be easily manipulated under natural conditions. Thus, the anuran vocal sac might be of great interest not only to herpetologists, but also to behavioural biologists studying communication systems.

## Highlights

• Our view of anuran communication has changed drastically during the last few decades.
• Anurans use diverse communication strategies often directly related to the vocal sac.
• Distinctive vocal sac shapes, sizes and colours suggest functional diversity.
• Evidence increases that the vocal sac also plays a role in chemical signalling.
• The anuran vocal sac may be of great interest for studies on communication systems.

Enormous signal diversity can be observed in animal communication systems. Physiological mechanisms are adapted over evolutionary time to generate and receive signals in various modalities (Bradbury & Vehrencamp, 2011). The courtship signals of acoustically communicating insects are produced by various stridulation processes (Gerhardt & Huber, 2002). In crickets, for example, the acoustic signals are generated by stridulation of the forewings, and the harp, a triangular anterior wing structure, acts as a sound oscillator and radiates an amplified narrow frequency band of the produced sound (Prestwich, Lenihan, & Martin, 2000). *Anolis* lizards rely almost entirely on visual signals; their colourful throat display advertises position, repels males and attracts female mates and habitat light conditions are suggested to promote signal diversity (Leal & Fleishman, 2004). Chemical signals of salamanders, pheromones produced by ‘mental’ chin glands, considerably increase a female's willingness to mate (Houck & Reagan, 1990; see also Vaccaro, Feldhoff, Feldhoff, & Houck, 2010). Similar signals can be found in anuran amphibians, although the predominant mode of intra- and intersexual communication is vocalization (Dorcas et al., 2010, Gerhardt and Huber, 2002, Ryan, 1985). It is reasonable to infer that advertisement calls of frogs, which attract females and signal readiness to defend territories to male opponents, were shaped over time through natural and sexual selection as by-products of breathing. However, during the last few decades, our view of anuran communication has broadened considerably. Anurans exhibit a striking diversity of communication strategies in the acoustic, visual, seismic and chemical domains, many of which are directly related to the so-called vocal sac, a morphological feature of most male frogs and toads (Liu, 1935).

This review aims to highlight the diversity of vocal sac morphology and function and its potential role as a ‘multimodal signalling tool’ in anuran communication. We further emphasize chemosignals as an additional and yet rarely investigated sensory modality in terrestrial anurans.

## The traditional view of the vocal sac

Prior to producing a call, frogs and toads fill their lungs with air. With the mouth closed and nostrils open, they inhale by lowering the floor of the mouth, creating a negative pressure, and air flows into the oral cavity. The nostrils subsequently close and the floor of the mouth is lifted to push the air into the lungs. The ventilation cycle is repeated until the lungs are sufficiently filled with air to facilitate call production. As amphibians lack ribs as well as a diaphragm and therefore are unable to passively inflate their lungs, the necessity of this procedure is apparent (Gans, 1970). Finally, to produce a call, an individual contracts its trunk muscles and air from the lungs flows via the vocal chords into the oral cavity. The airflow makes the vocal chords vibrate and a sound is produced. If frogs exhaled with each call, the lungs would rapidly be emptied and the calling individual would have to pause and again initiate the ventilation cycle. However, frogs may call for several minutes or even hours without pausing. Thus, for example, during the breeding season the New River tree frog, *Trachycephalus hadroceps*, is capable of producing up to 38 000 calls per night (Gaucher, 2002). This tremendous calling performance can only be achieved through an elastic skin pouch connected to the floor of the mouth which can store the air and use mechanical energy to push the air back into the lungs: the so-called vocal sac (Gans, 1973, Martin and Gans, 1972).

The anuran vocal sac probably evolved in response to selection for increasing calling efficiency (Bucher et al., 1982, Pauly et al., 2006). However, enabling a male to recycle air during calling is not the only way the vocal sac improves calling ability. Apart from fast lung reinflation it minimizes the loss of sound energy by decreasing the impedance mismatch between the frog's body cavity and its environment, increases the call rate and distributes sound waves omnidirectionally (Bucher et al., 1982, Pauly et al., 2006, Rand and Dudley, 1993). Calling with the mouth closed rather than open reduces the frequency range, hence narrows the bandwidth of a call and increases the intensity at the dominant frequency (Gridi-Papp, 2008). Thereby the vocal sac facilitates energetic effectiveness and acoustic conspicuousness, and is thereby also a species-specific advertisement signal.

## First hints of a more complex function

In frogs and toads, male advertisement calls play an important role in species recognition, mate choice, male spacing and territory defence (and see Toledo et al., 2014; reviewed in Wells, 1977, Wells, 1988). Thus, vocal sac movement has traditionally been seen as an epiphenomenon of call production (Dudley and Rand, 1991, Rand, 1988). Although the pulsation of the vocal sac can be interpreted as a necessary by-product of vocalization, there is increasing evidence that the visual properties of the vocal sac influence receivers and, combined with acoustic signals, form a fixed composite signal (sensu Partan & Marler, 2005). The vocal sac was first incorporated into the multimodal signal as a cue; however, colour variations and patterns could provide reliable indicators about the attributes of a sender (Gomez et al., 2009, Richardson et al., 2010; e.g. Vásquez & Pfennig, 2007) and might lead to an enhanced signal to noise ratio (Gerhardt & Schwartz, 2001).

Conspicuous white speckles on the otherwise black vocal sac of *Dendrobates pictus* clearly increase the visibility of a calling male to the human observer in a highly structured environment (Hödl, 1991). The first evidence that the pulsating vocal sac of a calling male frog might be used as a visual cue by conspecifics comes from the diurnal dart-poison frog, *Allobates femoralis* (Narins, Hödl, & Grabul, 2003). Male *A. femoralis* are highly territorial and show a stereotypic phonotaxis towards rival males calling within their territory. The phonotactic approach behaviour can be elicited by just the advertisement call and territory holders reliably locate the sound source in search of the intruder. When presented with species-specific advertisement call playbacks and a lifelike male model frog, conspecific males approached the speaker and showed no aggression towards the artificial intruder. Territory holders similarly showed no aggressive response when presented with exclusive vocal sac pulsations; however, temporally overlapping dynamic bimodal cues provoked fighting behaviour (Narins et al., 2003, Narins et al., 2005). Hence, conspecific vocalizations in *A. femoralis* elicit a phonotactic response and antiphonal calling but are not sufficient to provoke physical aggression. de Luna, Hödl, and Amézquita (2010) showed in a follow-up study that movement not only of the inflated vocal sac but also of a robotic frog model provoked territorial aggression in males of *A. femoralis*.

Similarly, the Kottigehar dancing frog, *Micrixalus kottigeharensis* (previously *Micrixalus saxicola*), a so-called foot-flagging species that performs leg waves during agonistic male interactions, only displays this behaviour in response to conspecific calls accompanied by vocal sac inflation. The visual cue was suggested to improve detection and discrimination of acoustic signals by making them more salient to receivers amid complex biotic background noise (Preininger et al., 2013).

In the East African stream frog, *Phrynobatrachus krefftii*, the conspicuous yellow vocal sac functions as a dynamic visual signal in male–male agonistic interactions even without calls being produced. The nonaudible vocal sac inflation used during male–male agonistic interactions might be a ritualized visual signal comparable to the colourful dewlaps of male *Anolis* lizards (Fitch and Hillis, 1984, Leal and Fleishman, 2004) or the striking red throat pouch in male great frigatebirds, *Fregata minor* (e.g. Juola, McGraw, & Dearborn, 2008).

Even in nocturnal species the vocal sac can be an important visual cue. Túngara frogs, *Engystomops pustulosus*, are visually sensitive at night (Cummings, Bernal, Reynaga, Rand, & Ryan, 2008) and females show a preference for advertisement calls synchronized with vocal sac inflation in video playbacks (Rosenthal, Rand, & Ryan, 2004) and robotic frog experiments (Taylor, Klein, Stein, & Ryan, 2008). Although the advertisement calls are sufficient for mate attraction, females assess multimodal stimuli during courtship. Likewise, females of the nocturnal European tree frog, *Hyla arborea*, prefer conspicuous colourful vocal sacs in addition to calls, suggesting that carotenoid-based vocal sac coloration might be a condition-dependent cue in this species (Gomez et al., 2009, Richardson et al., 2009). The availability of the vocal sac as a visual cue makes even an unattractive call with a slow call rate more appealing to females of *Hyla squirella*; however, the same result does not hold for the túngara frog, and hence added visual cues show differential modulation in female choice (Taylor, Klein, & Michael, 2011). In males of the explosively breeding common frog, *Rana temporaria*, the reflectance of the throat increases during the breeding season. The luminance of male throats, however, does not correlate with size, body condition or quality and has been suggested to be a nuptial visual cue for sex recognition in dense breeding aggregations (Sztatecsny, Strondl, Baierl, Ries, & Hödl, 2010).

Disentangling the influence that visual cues of the multimodal signal may have on receivers remains difficult. However, detailed investigations of isolated and combined signal properties show communalities and differences between species and taxa and lead to a better understanding of not only anuran communication, but generally also of signal perception in the animal kingdom.

Moreover, the movement of the vocal sac during calling can act as a vibrational or seismic cue. In the mostly ground-dwelling white-lipped frog, *Leptodactylus albilabris* (Lewis et al., 2001) vocal sac inflation against the ground produces substrate-borne vibrations and therefore additional seismic signals to overcome the acoustic noise of a heterospecific chorus (and see Caldwell et al., 2010, Cardoso and Heyer, 1995).

The examples given above highlight the diversity in vocal sac utilization as a visual cue or signal and yet suggest that several questions in regard to communication strategies remain to be answered.

## Vocal sac diversity suggests unexplored functions

**Figure 1:**
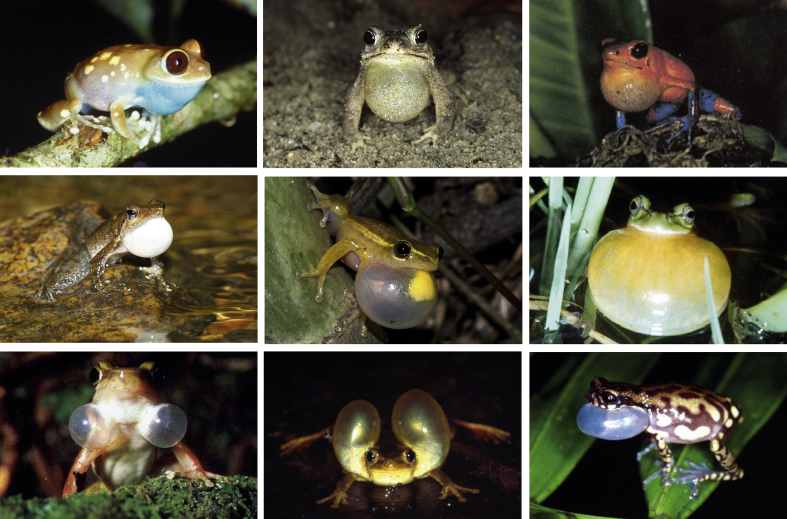
Examples to illustrate the striking vocal sac diversity of amphibians (from left to right). First row: *Leptopelis uluguruensis*, *Bufo granulosus*, *Dendrobates pumilio*; second row: *Micrixalus kottigeharensis*, *Hyperolius cinnamomeoventris*, *Hyla meridionalis*; third row: *Hylodes phyllodes*, *Trachycephalus coriaceus*, *Allophryne ruthveni*.

A striking diversity of vocal sac shape, size and colour can be found in frogs and toads (Fig. 1). A single subgular vocal sac seems to be the most commonly found vocal sac type (Wells, 2007); however, a similar shape does not imply a similar function.

Males of the European fire-bellied toad, *Bombina bombina*, have a subgular vocal sac, but produce sounds in the inspiratory phase. After every call their head hits the water surface thus generating waves which may be used in seismic communication (see Seidel, Yamashita, Choi, & Dittami, 2001). In *Bombina orientalis* calls are very soft, vocal sac inflation is not conspicuous and females use multimodal cues to approach a male in the water (Zeyl & Laberge, 2011). The density of water is similar to the density of the calling animal, which might lead to reduced vocal sacs or even to a lack of a vocal sac such as in the African clawed frog, *Xenopus laevis*, which calls underwater (Hayes & Krempels, 1986; e.g. Tobias et al., 2004). However, many species calling in water seem to have developed the opposite strategy by using two vocal sacs (e.g. *Pelophylax* spp., *Trachycephalus* spp.) which might facilitate floating in water and/or better airborne sound transmission (Wells, 2007). Such secondary sensory components can lead to risky consequences as recently shown in túngara frogs: water ripples produced by vocal sac inflation facilitate the localization of a calling male not only for conspecifics, but also for hunting bats, *Trachops cirrhosus* (Halfwerk, Jones, Taylor, Ryan, & Page, 2014).

**Figure 2:**
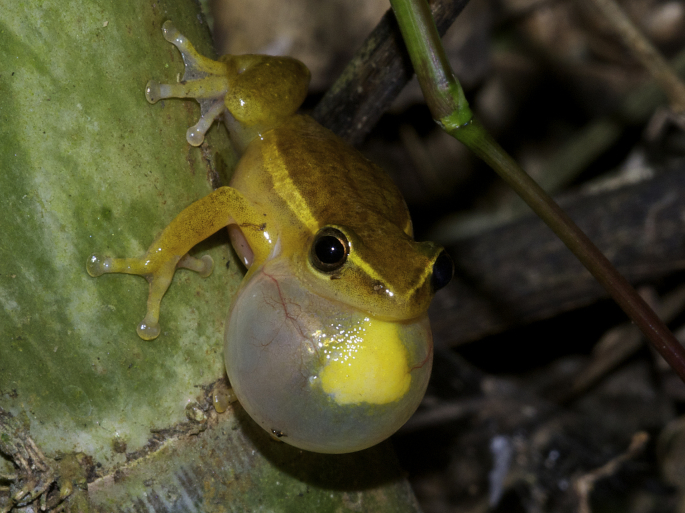
Male cinnamon-bellied reed frog, *Hyperolius cinnamomeoventris*, with inflated vocal sac. The gular gland and prominent blood vessels are clearly visible in the centre of the vocal sac.

**Figure 3:**
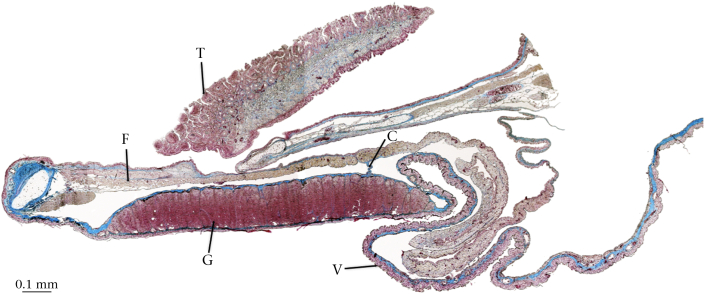
Histological section of the gular region of a male *Hyperolius riggenbachi* (sagittal, AZAN stain) including tongue (T) magnified by 40. The gular gland (G) tissue is clearly thicker than the surrounding vocal sac skin (V). A strand of connective tissue (C) joins the gular gland and the floor of the mouth (F).

Furthermore, there is increasing evidence that the vocal sac might also play a role in chemical signalling. In the family of African reed frogs (Hyperoliidae) there is substantial variation in body coloration, morphology and reproductive modes, but males of all reed frog species share a common feature: a prominent gular patch on the vocal sac, which is particularly conspicuous once the vocal sac is inflated (Fig. 2). Although the presence, shape and form of the gular patch are well-known diagnostic characters for these frogs, its function remained unknown until recently. Starnberger et al. (2013) demonstrated that the gular patch is a gland (Fig. 3) and produces species-specific volatile compound mixtures, which might be emitted while the male is calling. In the most species-rich hyperoliid genera (*Afrixalus*, *Heterixalus*, *Hyperolius* and *Phlyctimantis*) the proposed signal cocktails consist of 65 different compounds, whereas specific combinations of sesquiterpenes, alcohols and macrolides are correlated with species identity (Starnberger et al., 2013). Additionally, a surprisingly high contrast between the gular patch and the surrounding vocal sac skin makes the gland stand out from its background and might serve as a visual cue facilitating the localization of a male calling in dense vegetation (I. Starnberger, own observations). Thus, reed frogs might use a complex combination of acoustic, visual and chemical signals in species recognition and mate choice, so far not described in any other terrestrial anuran. Hyperoliids often call in mixed choruses with closely related species without an apparent spatial segregation (Lötters et al., 2004, Schiøtz, 1999), and multimodal signals might have evolved to avoid mismating and to facilitate navigation towards a conspecific mate in dense vegetation. In one genus of the family Hyperoliidae, Rödel, Kosuch, Veith, and Ernst (2003) described two mute species which may rely solely on chemical communication via their gular glands. Several further behavioural observations suggest chemical communication in a different social context. For example, in Fausto's button frog, *Cycloramphus faustoi*, males were observed to rest their vocal sac on egg clutches, possibly to transmit pheromones that influence larval development (L.F. Toledo, personal communication). In Canebrake frogs, *Aplastodiscus perviridis*, males rest their vocal sac on females suggesting pheromone transmission during courtship (Haddad, Faivovich, & Garcia, 2005).

Apart from the use of the anuran vocal sac, unimodal chemical signalling is widespread in amphibians but has received relatively little attention in the vast field of anuran communication (Starnberger, Preininger, & Hödl, 2014). The following section gives a brief overview of the chemical communication strategies of amphibians and of the use of chemosignals in aquatic and terrestrial environments.

## Chemical signalling in amphibians

Most amphibians have a biphasic life cycle, hence spend part of their life in water and because of their often thin and permeable skin (Duellman & Trueb, 1986) it seems reasonable that chemical signals might play an important role at least in the early stages of amphibian life histories (e.g. Jungblut et al., 2012, Schulte et al., 2011). In aquatic and terrestrial urodeles, there are many well-known cases of chemical communication. Different skin glands in newts (Hilton, 1902, Malacarne and Giacoma, 1986, Treer et al., 2013) and salamanders (Baird, 1951, Fontana et al., 2006, Noble, 1929, Truffelli, 1952) release chemosignals and are used to recognize and locate partners during courtship and mating as well as opponents in territorial defence (reviewed in Woodley, 2010). Whereas chemical communication is without doubt the dominant sensory modality in urodeles, little is known about the behaviour and communication of the vastly understudied amphibian group of caecilians (Eisthen & Polese, 2007; but see Reiss & Eisthen, 2008). A communication system based on chemosignals is suggested not only by their nocturnal and fossorial life (Waldman & Bishop, 2004), but also by the paired tentacle, an organ connected to the vomeronasal organ (Schmidt & Wake, 1990), and studies on waterborne chemical cues that attract conspecifics (Warbeck, Breiter, & Parzefall, 1996) and facilitate mate recognition (Warbeck & Parzefall, 2001).

In anuran amphibians, several tadpoles are able to detect chemical cues from predators (reviewed in Chivers & Smith, 1998; e.g. Pearl, Adams, Schuytema, & Nebeker, 2003) and chemical alarm stimuli from injured conspecifics (e.g. Hews, 1988; but also see Summey & Mathis, 1998). There are few reported cases of pheromones in adult aquatic frogs and toads. The silent and nocturnal tailed frogs, *Ascaphus truei*, live alongside noisy stream habitats, similar to foot-flagging species. Tailed frogs that were exposed to water previously containing reproductive males or females showed a preference for chemosignals of the opposite sex which suggests chemical mate recognition (Asay, Harowicz, & Su, 2005). Wabnitz, Bowie, Tyler, Wallace, and Smith (1999) found that female magnificent tree frogs, *Litoria splendida*, are attracted towards the male by ‘splendipherin’, an aquatic pheromone produced only by males in glands found on the head. Males of the mountain chicken frog, *Leptodactylus fallax*, secrete an aggression-stimulating peptide that provokes aggressive behaviour in males and has no effect on females (King, Rollins-Smith, Nielsen, John, & Conlon, 2005). In African clawed frogs (*Hymenochirus* sp.) females tested in Y-maze experiments showed a clear preference for water containing homogenized male postaxillary breeding glands or water previously containing live males (Pearl et al., 2000). The chemicals found in *L. splendida*, *L. fallax* and in *Hymenochirus* sp. are peptides and therefore can only be spread passively in water, but not as airborne chemical cues on land (Houck, 1998, Houck, 2009, Rajchard, 2005).

The above-mentioned studies demonstrate chemical communication as a relevant signal modality of anurans in the aquatic environment in varying social contexts.

A wide range of aquatic and also terrestrial anurans use chemical cues for navigation (Schulte et al., 2011, Sinsch, 1990) and predator detection (Flowers & Graves, 1997), which leads to the assumption that many species have the physiological and anatomical ability to produce and detect chemical signals also during terrestrial life phases (Byrne and Keogh, 2007, Woodley, 2010). During metamorphosis, the transition from water to land, anurans undergo changes in the olfactory system (Belanger & Corkum, 2009) strongly suggesting a difference in odorant access. Recent investigations concerning adaptations of the olfactory organ in one of the most basal anuran species (*A. truei*) denote the ability to receive airborne as well as waterborne odours (Benzekri & Reiss, 2012). Belanger and Corkum (2009) described that in adult anurans the nasal cavity is capable of aquatic and terrestrial chemosignal reception. Waterborne signals are detected by the vomeronasal organ, whereas airborne odorants are processed via the principal chamber.

As for volatile signal generation, many authors have speculated on the possible use of the skin glands present in males of many terrestrial anurans. Chemical communication via skin secretions seems to be most likely in a sexual context owing to a male's direct contact with the female during mating (i.e. amplexus; Lenzi-Mattos et al., 2005, Rödel et al., 2003; e.g. Thomas, Tsang, & Licht, 1993). But to the best of our knowledge, there are only two reported cases of pheromone communication in terrestrial anurans. In the Australian toadlet, *Pseudophryne bibronii*, males call when hidden in the leaf litter at night and secrete an odorous mucus produced by dorsal, axillary and postfemoral skin glands which is likely to help females in close-range mate localization and significantly influences male calling activity (Byrne & Keogh, 2007). In Mantellid frogs native to Madagascar, males have prominent femoral glands, which produce volatiles possibly acting as species-specific pheromones (Poth et al., 2013, Poth et al., 2012).

The use of pheromones in anuran species recognition and mate choice might be a widespread phenomenon, since all amphibians seem to possess a general predisposition towards the use of chemical cues (Waldman & Bishop, 2004) and since chemosignals can usually be produced at low cost (Hedin, Maxwell, & Jenkins, 1974).

Chemosignal transmission in amphibians has been suggested to occur predominantly in close-range interactions via direct contact or as short-range signals when released into water. One dispersion method is to release the odorants into a self-generated current (Bradbury & Vehrencamp, 2011) as performed by newts when flicking their tail during underwater courtship. Similar dispersion might be achieved by vocal sac pulsations which potentially fan out volatiles produced by a gular gland or even discharge a puff with each call. Without doubt, constraints such as wind could easily disturb the distribution of airborne chemical cues and therefore their localization by receivers. However, environmental conditions in the breeding swamps of calling reed frogs (i.e. high temperatures and humidity as well as elevated perching sites) would facilitate (at least) airborne short-range transmission. Thus, the inevitable vocal sac movement during calling could, apart from its visual signal content, serve to disperse chemical signals. Frogs and toads may possess more sophisticated chemical communication strategies than science has previously given them credit for, in addition to or instead of acoustic signals. To date, chemical communication in anurans has been overlooked by most studies (Belanger and Corkum, 2009, Houck, 1998, Houck, 2009, Waldman and Bishop, 2004, Woodley, 2010), probably because of the presence of more conspicuous signals (e.g. acoustic and visual) or overly costly and elaborate analyses necessary to investigate pheromones.

## Guidelines for future research

Females as well as males of many anuran species are confronted with the challenge of detecting, locating and discriminating conspecific males in breeding aggregations (Bee and Micheyl, 2008, Richardson and Lengagne, 2010) or environments that hamper propagation and transmission of acoustic signals (Boeckle et al., 2009, Kime et al., 2000, Wells and Schwartz, 1982). Generally, signals should evolve to maximize the signal to noise ratio and to improve efficacy. Any signal detectability depends on signal design, environmental conditions or the capacity of the medium used to transmit a signal and of course on the receiver's sensory system (Endler, 1992, Endler, 1993, Shannon, 1948). The use of multiple signal modalities, where each modality increases efficacy under specific conditions, can enhance signal conspicuousness in a variety of sensory modes (Ryan & Keddy-Hector, 1992). Misinterpreting or missing a signal is probably related to high costs. Thus a selective pressure could lead to the use of additional or alternative sensory signal modalities (Krebs & Dawkins, 1984). Furthermore, the combination of an unattractive acoustic signal and an unattractive visual signal can result in a highly attractive multimodal signal (Taylor & Ryan, 2013).

The anuran vocal sac seems likely to play a role in multimodal signalling in many more species than previously thought. Future studies should focus on describing and empirically testing the diverse functions of the vocal sac in different anuran taxa. Furthermore, there might be patterns of signal modalities and morphological features (e.g. vocal sac size) related to certain environmental conditions or habitat features (diurnal versus nocturnal, arboreal versus terrestrial versus aquatic, cue received by males, females and/or heterospecifics). To shed light on signal function, more across-species comparisons of how single and combined signal components influence receivers are needed. Furthermore, detailed descriptions of the conditions in which signals are produced are essential to draw more general conclusions on vocal sac signal function. Finally, we should question the constraints of different modalities and therefore the costs imposed by sexual or natural selection, to explain differences and similarities of this multimodal communication tool. Moreover, the majority of descriptions of amphibian glands involved in chemical courtship and mating signals lack information on the structure of the glands. This information would allow for more specific hypotheses on the origin and function of the glands described.

### What Amphibians Have to Offer Research on Chemical Communication

Within vertebrates, amphibians possess a rather basal nervous system leading to a mostly stereotypic behaviour (but see for example Pašukonis et al., 2014, Schulte et al., 2011). Amphibians in general and frogs in particular are excellent model organisms to experimentally investigate communication strategies both in the laboratory and under natural conditions, as they are tolerant to disturbance by observers and can be easily manipulated (Hirschmann and Hödl, 2006, Klein et al., 2012, Narins et al., 2003, Taylor et al., 2008).

Amphibians show a vast diversity in reproductive modes (e.g. Hödl, 1990) and owing to their adaptations to aquatic and terrestrial habitats we can expect insights into the evolution of chemical signals in water and on land (reviewed in Woodley, 2014).

To date, a number of amphibian pheromones have already been identified (Kikuyama et al., 2002, Poth et al., 2012, Toyoda et al., 2004, Wabnitz et al., 1999), but in comparison to the vast number of amphibian species worldwide (nearly 7300), these are probably only the tip of the iceberg.

## Conclusion

The vast diversity of anuran vocal sacs suggests a multitude of different functions in intra- and intersexual communication shaped over evolutionary time by natural and/ or sexual selection. The modalities used in addition to or instead of acoustic signals range from seismic and visual to chemical. The vocal sac, a single morphological feature, has the potential to generate multimodal signals simultaneously or sequentially. Signals of the same modality might be directed towards and perceived by one or more conspecifics and heterospecifics. Furthermore, even signals shaped under similar evolutionary constraints may influence receivers differentially. Thus, the anuran vocal sac might be of great interest not only to herpetologists, but also to behavioural biologists investigating unimodal or multimodal communication in the acoustic, visual or chemical domains. Amphibians are highly suitable organisms to study chemical unimodal and multimodal signalling in a basal system. The focus of chemical signal research in amphibians, however, used to be mainly on urodeles. In anurans the salient acoustic and visual signals might to a certain extent have deflected our attention from equally fascinating chemical signalling strategies. Possibly, terrestrial anurans might have lost once-present chemical signals owing to the evolutionary success of acoustic communication. In some species acoustics might not have been sufficient, and thus chemical signals were ‘rediscovered’ and incorporated into a multimodal signal or in some species even fully replaced acoustic signals.

In this review we hope to have stimulated researchers to investigate potential chemical signals in anuran communication systems and to recognize the vocal sac as a unique multimodal signalling tool.
